# Corrections of Dental Anomalies in the Maxillary Incisors and Their Influence on Perceived Smile Esthetics: A Systematic Review

**DOI:** 10.3390/bioengineering12030262

**Published:** 2025-03-06

**Authors:** Nessa Rose McGarty, Caterina Delre, Carlo Gaeta, Tiziana Doldo

**Affiliations:** 1 Unit of Orthodontics, Department of Medical Biotechnologies, University of Siena, 53100 Siena, Italy; nessa.mcgarty@student.unisi.it (N.R.M.); caterina.delre@student.unisi.it (C.D.); 2 Unit of Periodontology, Endodontology and Restorative Dentistry, Department of Medical Biotechnologies, University of Siena, 53100 Siena, Italy; carlo.gaeta@unisi.it

**Keywords:** dental anomalies, tooth abnormalities, esthetics, dental, restorative restoration, microdontia, incisor agenesis, peg shaped laterals, maxillary incisors

## Abstract

Introduction: Dental anomalies present a significant challenge to clinicians due to their impact on both dental function and esthetics. The correction of these anomalies plays a critical role in improving the quality of life of our patients, highlighting the importance of this restorative work. Objective: The purpose of this systematic review is to assess the techniques used to restore various dental anomalies, and their subsequent esthetic impact on the overall dentition. Methods: Inclusion criteria consisted of restorative rehabilitations of the permanent dentition in non-syndromic patients with dental anomalies of morphology, structure, size, and number in the maxillary incisors. Exclusion criteria included surgical rehabilitation techniques, endodontic treatments, and anomalies of the primary dentition. The medical literature was systematically searched (Pubmed, PMC, Embase, Cochrane Central Register of Controlled Clinical trials, Scopus and Google Scholar) to identify all relevant articles reporting data regarding the chosen anomalies. ROBINS–I was used to assess the risk of bias tool, and the results were tabulate due to data heterogeneity. Results: Of the 1821 analyzed articles, 46 articles met the inclusion criteria, and were chosen to go through the final review procedure. Of the selected articles, 3 investigated amelogenesis imperfecta and dentinogenesis imperfecta, 1 analyzed conoid teeth, 1 considered hypodontia (other than MLIA), 3 concerned microdontia (excluding peg laterals and conoid teeth), 10 evaluated peg-shaped laterals, 2 investigated talon cusps and geminated teeth, 15 were regarding maxillary lateral incisor agenesis, and 11 papers were related to the perception of anomalies. Conclusions: Pre-visualization using Digital Smile Design, a treatment plan encompassing minimally invasive restorations, and using a multidisciplinary approach among practitioners helps the anomalous patient achieve the best possible esthetic result.

## 1. Introduction

The transformative power of an esthetic smile has proven to significantly enhance social perceptions, professional success, overall health, and emotional well-being [1].

However, the esthetics of a smile are significantly altered when we have anomalies of the dentition, e.g., if we are missing teeth, if we have extra teeth, or if our teeth are of an unusual morphology.

Dental anomalies present a significant challenge to clinicians due to their impact on both dental function and esthetics, which in most cases requires an interdisciplinary approach to finding solutions [2].

An anomaly is defined as anything that deviates from the accepted standard, normal or expected [3]. The objective of our restorative treatments is to correct dental anomalies and restore the affected dentition to a form that aligns with the established esthetic and functional standards.

Studies have shown that dental anomalies, on a parasocial relationships level [2], often cause psychological stress and diminished quality of life for individuals, which further highlights the need for and importance of effective esthetic restorative treatments.

While the prevalence of anomalies differs depending on factors such as gender and race, clinicians agree that no community is absolved of the occurrence of dental anomalies themselves.

Our systematic review aims to investigate how we go about restoring these anomalies, and what is needed to make our smile the most esthetic possible, in order to enhance our patients’ quality of life while also restoring appearance and functionality.

## 2. Rationale

As far as we are aware, in the current literature, there is no study to compare multiple dental anomalies and their restorative options, much less the esthetic outcome of these restorations in the anomalous dentition. Our reasoning behind covering multiple anomalies is that anomalies often occur simultaneously.

There are multiple studies conducted on restorative options to treat certain dental anomalies, such as lateral incisor agenesis [4,5,6,7]. However, there is very limited literature on the esthetic outcome of said restorations, nor the framework used to achieve the most esthetic restorations possible.

## 3. Objective

This systematic review aims to collate the available information on various dental anomalies of size, structure, number, and morphology of the maxillary incisors and lay out a clear framework for the various conservative rehabilitation techniques that can be performed to restore optimal esthetic appearance in conjunction with patient satisfaction.

We will systematically synthesize the relevant literature, providing evidence-based recommendations for clinical practice to identify areas where further research is needed.

The purpose of this review is to support dental professionals planning cases involving dental anomalies, ensuring they can decide the best available treatment option for their patient, with the most relevant literature.

### 3.1. Choice of Anomalies

After our initial literature search, we decided to combine multiple anomalies of size, structure, number, and morphology. This was due to the extensive studies showing the occurrence of more than one anomaly in an already anomalous dentition.

Multiple studies have shown that unilateral agenesis is often linked to the contralateral tooth’s corresponding microdontia [8,9]. There is also a proven link between agenesis and root morphological changes, such as short roots [10]. A link has also been found between hypodontia and its association with other anomalies [11]. Dentinogenesis imperfecta can also be associated with the short root anomaly [12].

There has been no review encompassing multiple anomalies of this magnitude, despite this evident overlap in its occurrence, necessitating the need for our review.

Chosen anomalies can be seen in Table 1.

### 3.2. Prevalence

Obtaining accurate statistics on the prevalence of the various dental anomalies was challenging due to their variability across different races and countries. Comprehensive studies have only been conducted in select nations, such as this study conducted in Turkey [14].

Therefore, in the following infographic, it is important to remember there is a discrepancy between the various ethnic backgrounds.

## 4. Materials and Methods

### 4.1. Protocol and Registration

The review is in the process of being registered on PROSPERO (International Prospective Register of Systematic Reviews). Prospero registration number ID, CRD42024532371.

### 4.2. Eligibility Criteria

We included as many of the relevant anomalies related to the maxillary incisors in adult patients with permanent dentition as possible, including anomalies of structure, size, number, and morphology.

Studies were grouped according to their anomaly, and the relevant restorative techniques and esthetic outcomes were noted.

See below (Table 2) for a detailed table of inclusion and exclusion criteria according to our PICO criteria

### 4.3. Information Sources

We did not restrict searches to particular results because of the diversity of available studies. However, all studies reporting on dental anomalies, esthetics, and restorative rehabilitation techniques were included to collate all relevant treatment methods, which were later screened and filtered according to our inclusion and exclusion criteria.

The search for available literature was performed according to the PRISMA guidelines [19] by searching Pubmed, Google Scholar, Embase, Cochrane Library, and Scopus databases until 15 May 2024.

### 4.4. Search Strategy

The search strategy consisted of two separate searches, the first relating to the relevant anomalies and restorative restorations and the second to the relevant anomalies and esthetic dentistry. The search also incorporated [MeSH] terms on the relevant platforms.

Filters were placed for the search results including the year of publication and the language. The limit was placed in terms of year of publication—only articles within the last 20 years were eligible. Results were filtered depending on the language—English. No additional filters were applied to the search results.

The search strategies were performed with consultation from an experienced research librarian, ensuring the identification of keywords and possible combinations to identify the most relevant studies for our subject topic.

#### 4.4.1. Search 1 (Restorative Restoration)

##### Pubmed

(Dental anomalies OR “Tooth Abnormalities” [Mesh] OR amelogenesis imperfecta OR dentinogenesis imperfecta type 2 OR macrodontia OR microdontia OR short roots OR “Tooth, Supernumerary” [Mesh] OR incisor agenesis OR talon cusp OR Fused Teeth OR tooth gemination OR Dens in dente OR dens invaginatus OR peg-shaped laterals OR conoid teeth) AND maxillary incisors AND restorative restoration.

##### Google Scholar and Cochrane Library

(Dental anomalies) OR (amelogenesis imperfecta) OR (dentinogenesis imperfecta type 2) OR (macrodontia) OR (microdontia) OR (short roots) OR (supernumerary teeth) OR (incisor agenesis) OR (talon cusp) OR (tooth fusion) OR (tooth germination) OR (dens invaginatus) OR (peg-shaped laterals) OR (conoid teeth) AND (maxillary incisors) AND (esthetic dentistry).

##### Embase

(“dental anomalies”/exp OR “tooth malformation”/exp OR “amelogenesis imperfecta”/exp OR (“amelogenesis” AND “imperfecta”) OR “amelogenesis imperfecta” OR ((dental* OR tooth OR teeth) NEAR/2 (anomal* OR malformat* OR gemin* OR conoid*)) OR (“dentinogenesis” AND “imperfecta”) OR “dentinogenesis imperfecta type 2” OR “DI-2” OR “hereditary opalescent dentin” OR “macrodontia”/exp OR “macrodont*” OR “microdontia”/exp OR “microdont*” OR (short* AND “root*”) OR “supernumerary tooth”/exp OR “supernumerary tooth” OR “supernumerary teeth” OR (incisor* NEAR/2 agenesis) OR “talon cusp”/exp OR “talon cusp*” OR (“fused” AND “teeth”) OR “fused teeth” OR “invaginated tooth”/exp OR “dens invaginatus” OR “dens in dente” OR ((“dens” OR tooth OR teeth) NEAR/2 “invaginat*”) OR (“peg” AND “shap*” AND lateral*)) AND ((“maxilla”/exp OR “maxilla*”) AND (“incisor”/exp OR “incisor*”)) AND “restor*”.

##### Scopus

(TITLE-ABS-KEY ((amelogenesis AND imperfecta) OR “amelogenesis AND imperfecta”) OR TITLE-ABS-KEY (((dental* OR tooth OR teeth) AND (anomal* OR malformat* OR gemin* OR conoid*))) OR TITLE-ABS-KEY ((dentinogenesis AND imperfecta) OR “dentinogenesis AND imperfecta AND type AND 2′ OR “di-2” OR “hereditary AND opalescent AND dentin” OR macrodont* OR microdont*) OR TITLE-ABS-KEY ((short* AND root*) OR “supernumerary AND tooth” OR “supernumerary AND teeth” OR (incisor* AND agenesis) OR “talon AND cusp” OR (fused AND teeth) OR “fused AND teeth” OR “dens AND invaginatus” OR “dens AND in AND dente” OR ((dens OR tooth OR teeth) AND invaginat*) OR (peg AND shap* AND lateral*)) AND TITLE-ABS-KEY ((maxilla* AND incisor*)) AND TITLE-ABS-KEY (restor*)).

#### 4.4.2. Search 2 (Esthetic Dentistry)

##### Pubmed

(dental anomalies OR “Tooth Abnormalities” [Mesh] OR amelogenesis imperfecta OR dentinogenesis imperfecta type 2 OR macrodontia OR microdontia OR short roots OR “Tooth, Supernumerary” [Mesh] OR incisor agenesis OR talon cusp OR Fused Teeth OR tooth gemination OR Dens in dente OR dens invaginatus OR peg-shaped laterals OR conoid teeth) AND maxillary incisors AND “Esthetics, Dental” [Mesh].

##### Google Scholar and Cochrane Library

(dental anomalies) OR (amelogenesis imperfecta) OR (dentinogenesis imperfecta type 2) OR (macrodontia) OR (microdontia) OR (short roots) OR (supernumerary teeth) OR (incisor agenesis) OR (talon cusp) OR (tooth fusion) OR (tooth germination) OR (dens invaginatus) OR (peg-shaped laterals) OR (conoid teeth) AND (maxillary incisors) AND (restorative restoration).

##### Embase

(“dental anomalies”/exp OR “dental anomalies” OR “tooth malformation”/exp OR “tooth malformation” OR ((“amelogenesis”/exp OR “amelogenesis”) AND “imperfecta”) OR “amelogenesis imperfecta”/exp OR “amelogenesis imperfecta” OR ((dental* OR tooth OR teeth) NEAR/2 (anomal* OR malformat* OR gemin* OR conoid*)) OR ((“dentinogenesis”/exp OR “dentinogenesis”) AND “imperfecta”) OR “dentinogenesis imperfecta type 2′ OR “di-2” OR “hereditary opalescent dentin” OR “macrodontia”/exp OR “macrodontia” OR “macrodont*” OR “microdontia”/exp OR “microdontia” OR “microdont*” OR (short* AND “root*”) OR “supernumerary tooth”/exp OR “supernumerary tooth” OR “supernumerary teeth”/exp OR “supernumerary teeth” OR (incisor* NEAR/2 agenesis) OR “talon cusp”/exp OR “talon cusp” OR “talon cusp*” OR (“fused” AND (“teeth”/exp OR “teeth”)) OR “fused teeth”/exp OR “fused teeth” OR “invaginated tooth”/exp OR “invaginated tooth” OR “dens invaginatus”/exp OR “dens invaginatus” OR “dens in dente”/exp OR “dens in dente” OR ((“dens” OR tooth OR teeth) NEAR/2 “invaginat*”) OR ((“peg”/exp OR “peg”) AND “shap*” AND lateral*)) AND (“maxilla”/exp OR “maxilla” OR “maxilla*”) AND (“incisor”/exp OR “incisor” OR “incisor*”) AND (“dental procedure”/exp OR “dental procedure” OR ((aesthetic* OR esthetic*) NEAR/2 (dentistry OR dental))) AND ([article]/lim OR [article in press]/lim OR [conference paper]/lim OR [conference review]/lim OR [data papers]/lim OR [editorial]/lim OR [letter]/lim OR [note]/lim OR [review]/lim OR [short survey]/lim).

##### Scopus

(TITLE-ABS-KEY ((amelogenesis AND imperfecta) OR “amelogenesis AND imperfecta”) OR TITLE-ABS-KEY (((dental* OR tooth OR teeth) AND (anomal* OR malformat* OR gemin* OR conoid*))) OR TITLE-ABS-KEY ((dentinogenesis AND imperfecta) OR “dentinogenesis AND imperfecta AND type AND 2′ OR “di-2” OR “hereditary AND opalescent AND dentin” OR macrodont* OR microdont*) OR TITLE-ABS-KEY ((short* AND root*) OR “supernumerary AND tooth” OR “supernumerary AND teeth” OR (incisor* AND agenesis) OR “talon AND cusp” OR (fused AND teeth) OR “fused AND teeth” OR “dens AND invaginatus” OR “dens AND in AND dente” OR ((dens OR tooth OR teeth) AND invaginat*) OR (peg AND shap* AND lateral*)) AND TITLE-ABS-KEY ((maxilla* AND incisor*)) AND TITLE-ABS-KEY (((aesthetic* OR esthetic*) AND (dentistry OR dental)))).

### 4.5. Selection Process

Title and abstract screening were performed double-blindly using Rayyan [20] by two authors (N.M.G., C.D.) to select articles for further reading and full-text retrieval. Duplicate papers were eliminated, and the authors selected the remaining papers independently. Authors (N.M.G., C.D.) then discussed papers selected for inclusion, and any disagreements were resolved by discussion and adhering strictly to the inclusion and exclusion criteria.

Full texts of the relevant papers were further examined to assess eligibility for data extraction. A manual search of the bibliographies in each article was also performed to identify any additional relevant papers.

### 4.6. Data Items and Data Collection Process

After discussion and agreement on the final articles, articles were divided evenly between authors (N.M.G., C.D.). Papers were divided into a series of subcategories according to their anomalies (Amelogenesis imperfecta + Dentinogenesis imperfecta, Conoid teeth, Hypodontia, Micro + Macrodontia, MLIA, Peg-shaped laterals, Talon cusp + Geminated teeth) and a separate category was made that was just dedicated to perception. Many of these anomalies can fall into multiple categories, i.e., conoid teeth and peg-shaped laterals in microdontia, MLIA into hypodontia; thus, it was decided that it would be placed into the relevant subcategories only if the anomaly was specifically stated in the title, otherwise it was placed in the broader category.

Data collected included a detailed description of the relevant intervention performed under the categories according to our PICO criteria; population, intervention-restorative rehabilitation, comparison, and, if applicable, a description of the esthetic parameters used to assess the outcome of the final restoration, under outcome-esthetics.

### 4.7. Study Risk of Bias Assessment

For this restorative treatment outcome review, we considered the quality of reported outcomes and rehabilitation to be crucial, so we selected ROBINS–I [21] as our risk of bias assessment tool. A breakdown of our critical appraisal results can be found below at Table 3.

### 4.8. Synthesis Methods and Effect Measures

This systematic review’s articles were so diverse that conducting a meta-analysis was not practical. Rather, a qualitative synthesis was carried out by contrasting the restorative techniques with their esthetic results based on the groups that were assessed.

## 5. Results

### 5.1. Study Selection

Of the 1821 analyzed articles, 46 articles met all of our criteria [22,23,24,25,26,27,28,29,30,31,32,33,34,35,36,37,38,39,40,41,42,43,44,45,46,47,48,49,50,51,52,53,54,55,56,57,58,59,60,61,62,63,64], and were chosen to go through the final review procedure. Among the chosen articles, 3 investigated amelogenesis imperfecta and dentinogenesis imperfecta, 1 analyzed conoid teeth, 1 considered hypodontia (other than MLIA), 3 concerned microdontia (excluding peg laterals and conoid teeth), 10 evaluated peg-shaped laterals, 2 investigated talon cusps and geminated teeth, 15 were regarding maxillary lateral incisor agenesis, and 11 papers were related to the perception of anomalies.

See Figure 1 Prisma flow diagram and Table S1 (Supplementary File) displaying the detailed PRISMA checklist for a detailed breakdown.

### 5.2. Study Characteristics

We split the studies into subgroups depending on their anomaly and also included another subgroup just for perception. Overlapping anomalies were put into the category that had the most in-depth information about the anomaly. In each paper, we extracted information on the study design, population, intervention/restorative technique, comparison, and outcome/esthetic impact.

### 5.3. Results of Individual Studies

A meta-analysis was not feasible due to the high level of heterogeneity among the included studies. We decided to use study tables (See below Table 4) to display our results.

## 6. Discussion

### 6.1. Summary of Evidence

#### 6.1.1. Amelogenesis Imperfect and Dentinogenesis Imperfecta

The studies highlight various interventions tailored to the specific conditions and needs of the patients. The prosthodontic pathway for individuals with tooth structure abnormalities [66] emphasizes minimal preparation to preserve tooth tissue. The use of veneers is particularly noteworthy, due to the difficulty with bonding of these structural anomalies [67], as it offers an esthetically pleasing and tissue-conservative alternative to crowns, which may not be suitable for severely affected teeth. Veneers are indicated in the anterior dentition, particularly if direct composite bonding has previously been attempted and proved unsuccessful [68].

Conventional crowns are successful in patients with AI and have been proven to be both durable and predictable, but with the marked disadvantage of being destructive of tooth tissue [69]. The risks in crown placement of AI patients include failure of cementation, material fracture, caries and endodontic treatment [70], all of which are also common complications of crowning teeth in the non-anomalous population.

In the case of minimally destructive management of AI and hypodontia [23], a combination of bleaching, gingivectomy, composite bonding, and cantilever bridges on canines were performed. This multifaceted approach not only addressed the esthetic concerns but also maintained the integrity of the remaining tooth structure. The emphasis on minimal invasiveness is evident in the choice of bleaching and composite bonding over more invasive procedures such as traditional veneers or bridges.

Similarly, bleaching was also used in another minimally invasive case [24] along with micro abrasion, which had a marked effect on the esthetic outcome. Bleaching is known to work well in cases of AI and fluorosis [71], and in these cases, at-home bleaching with custom trays of Carbamide Peroxide 15% for 2 weeks at home overnight is advised. Toothpaste containing 5% potassium nitrate on alternate nights is a good recommendation for those with AI and DI due to the increased sensitivity due to the nature of this anomaly [72].

The sequential use of crown lengthening, home bleaching, micro abrasion, and veneers demonstrates a comprehensive approach to improving both the esthetics and function of the dentition [24]. The use of a silicon key as a guide for veneer preparation underscores the importance of precision in achieving optimal esthetic outcomes.

#### 6.1.2. Conoid Teeth

Not as many articles were retrieved on this topic, since the majority of conoid teeth literature we found in this area either involved an endodontic treatment or was extracted and therefore excluded from our results due to our criteria.

This case [25] used a mix of direct and indirect restorative methods to produce the most esthetic result. The minimally invasive techniques followed in the conservative preparation of the porcelain veneer of the conoid tooth are in line with what the biomimetic approach [73].

The treatment protocol in the article involves esthetic crown lengthening, bleaching, conservative preparations for ceramic laminate veneers, and direct composite restorations. The step-by-step process, including the use of silicon indices, ensures precise and controlled tooth reduction, preserving tooth structure while achieving optimal esthetic integration.

#### 6.1.3. Hypodontia

The article emphasizes the need for thorough diagnostics and careful planning in managing hypodontia [26]. Dental technicians contribute significantly during the diagnostic phase by fabricating study casts, diagnostic wax-ups, and mock-ups, which aid in visualizing the final outcome and planning the treatment steps effectively [49,74].

The various restorative options include removable prostheses, interim fixed restorations, and definitive fixed restorations. The practical considerations for designing these anterior restorations involve understanding the esthetic and functional principles, such as the use of suitable abutment teeth, optimizing pontic design, and ensuring proper occlusion [75]. The article discusses the challenges in achieving these principles and provides practical solutions, such as modifications to abutment teeth. Canines that have been orthodontically repositioned are likely to rotate when relapsing [44]. Consequently, a cantilever lateral incisor bridge that is fixed to a canine may relapse.

Since a single restoration can be advantageous and have a high success rate, an alternative is to replace both lateral incisor teeth with a properly designed bridge from both maxillary central incisors. The mesial surface contact area of the pontic can be adjusted to simply overlap onto, but not bond to, the palatal surface of the neighboring central incisor tooth in order to lessen the possibility of rotational relapse, which would impact the lateral incisor pontic. The central incisor can still be adequately cleaned to lower the risk of caries, and this should be enough to stop outward rotation. Additionally, if the orthodontic treatment had closed a gap between the central incisor teeth, this design guarantees that the teeth will not relapse significantly [26].

#### 6.1.4. Microdontia and Macrodontia

All three studies [27,28,30] emphasize the importance of accurate diagnostics and treatment planning. While Bolton analysis was a key diagnostic tool used in two of the selected studies [28,30], the third study introduces an alternative method for calculating ideal tooth sizes, based on the mandibular central incisor, which is less variable in size [27].

The studies employ a range of minimally invasive techniques. The first focuses on a comprehensive interdisciplinary approach [30], while the second combines bleaching, enameloplasty, and bonding to enhance esthetics [28]. The third highlights early restoration during orthodontic treatment; a few more advantages include lowering the likelihood of having insufficient room to complete the final restoration and using the tooth as a temporary restoration while the rest of the treatment is being performed to assess its size and color, which can then be readjusted [27].

All studies aim to achieve superior esthetic outcomes by considering multiple factors such as tooth size, shape, and gingival margins. The emphasis on minimally invasive techniques and thorough diagnostics is consistent across all studies, ensuring that the final restorations are both functional and visually pleasing.

#### 6.1.5. Peg Laterals

These articles were mostly case reports. The main decision in the case of a peg lateral tooth is whether to pull the tooth and close the space or restore the original anomalous peg lateral [53]. To help decide the best option for each patient and their individual needs, a pre visualisation tool such as the Digital Smile Design was implemented in many cases [47,65].

If the peg lateral is retained, the next decision to be made is the type of restoration. Two main options are available and used: indirect method such as a veneer, or the direct method of composite bonding. However, a nice compromise was made in one of the cases, with a very good esthetic outcome, following the golden proportions, with a mix of both options in the form of a componeer [39].

When the indirect option is used, creating space for the prosthetic is vitally important. In the cases that used veneers, orthodontic movement was necessary to create adequate space for these restorations [51,71]. The space-creation technique uses interdental elastics to redistribute the patient’s diastemas, and it means there is no need for orthodontic appliances. However, caution must be taken as this technique can only be used for very short periods of time, if left for extended periods, even weeks, it can cause irreversible damage to the periodontal ligament [72].

For the direct option, composite bonding along with the putty technique was predominantly used, with very good esthetic outcomes [56,62]. A low interproximal space meant direct bonding was used in this case; conversely, in the same patient, veneers were chosen to mask the high interproximal space on the other peg-shaped incisor [65].

In terms of aestetic parameters, one study used the golden proportion [39] and another used the RED proportion [51].

#### 6.1.6. Perception

The vast majority of the articles retrieved involved an observational study in which a smile was digitally altered, and involved the analysis of certain key parameters. Similarly, the population chosen for most of the studies was related to the difference in perception between dental professionals and laypeople.

Unsurprisingly, orthodontists were seen to be the most observant and critical of alterations from the so called “ideal smile” [56,60], which has been proven repeatedly in the literature [60]. An interesting observation was the fact that a person’s own dental history was shown to have an impact on their perception of ideal smile esthetics, with the hypodontia patients in this study preferring lateral incisors considerably longer than the other participants in the study [64].

A slight midline discrepancy was the least observable characteristic in multiple studies.

The smile line was also a matter of contention; the medium smile line was regarded as the most esthetic, in this study, whereas a high smile line with diastemas was seen as the least esthetic [56].

In the studies that compared space closure versus space opening, both showed a preference towards space closure, perceiving it as a more esthetic outcome [61,76,77]. Only the dental professionals perceived a difference in the gingival margins as important in the overall esthetics, whereas laypeople actually perceived it as less attractive [39,53].

Asymmetric situations were seen as unattractive for both laypeople and dental professionals [60].

The golden proportion was not always regarded as the most esthetically pleasing choice, according to studies that used it. This has also been observed in the literature [48,75]. It can still prove to be a useful guide for the proportions, but as this study showed, it proved it is a range depending on the individual and their smile characteristics [57,61].

#### 6.1.7. Maxillary Lateral Incisor Agenesis

The selected articles supported the findings not only in the dental literature, but also in the other studied anomalies.

A very important factor in many of the orthodontic space-closure cases highlighted the importance of a light-colored canine. Canines are naturally darker in color with respect to their incisor counterparts due to their structural composition. This difference in color proved to be hugely important for the esthetic appearance, not only for dental professionals, but also laypeople [30,33]. This highlights the importance of pre-restorative bleaching in cases of orthodontic space closure.

Another frequent finding was in terms of the shape of the replacement canine. Pointed canines, those with a tip, were perceived as highly unesthetic. Many participants rated smiles with canine substitution as attractive only if the substituted canine approximated the lateral incisor in terms of shape, color, and gingival margin [30,33,45]. This sentiment was also backed up in terms of shape and their width, as the narrow canines were consistently perceived as more attractive [33].

Gingival margins were deemed very important for dental professionals, especially the orthodontists, but not perceived as important for laypeople [33]. This reiterates the earlier finding of dental professionals being more critical in their judgements [56]. Canine gingival height was the most attractive 0.5 mm below the gingival margin of the maxillary central incisor. A good solution for creating this optimal margin, which is not naturally occurring in cases of orthodontic space closure, is extruding the canine and intruding the first premolar in order to obtain ideal gingival architecture [32].

#### 6.1.8. Talon Cusp and Geminated Teeth

The chosen articles all used a conservative approach, with gradual cuspal grinding, in some cases performed in multiple sessions to be as conservative as possible and to allow the deposition of reparable dentin for pulpal protection due to the unusual anatomy in these teeth. Both cases were followed up with composite bonding for optimal esthetics [55,56].

### 6.2. Limitations

There was an evident lack of standardization to measure patient satisfaction in terms of the esthetic outcome of the restorative interventions.

Our inclusion and exclusion criteria meant that some of our retrieved articles had to be excluded due to surgical or endodontic interventions; for example, the anomalies of dens invaginatus, dens evaginatus, supernumerary teeth, as well as short root anomalies.

A nice technique that involves retaining the deciduous teeth to be restored in cases of MLIA was also excluded due to our criteria, and a lot of the literature on anomalous dentition was related to primary dentition.

### 6.3. Future Considerations

A similar systematic review regarding surgical techniques in the treatment of an anomalous dentition would also provide significant value to the field.

### 6.4. Clinical Implications

This systematic review can be used as an easy reference for determining the best restorative technique for each anomaly of the dentition.

## 7. Conclusions

As demonstrated, a comprehensive multi-disciplinary treatment plan, between the orthodontist, restorative dentist, and general dental practitioner, taking into account the individual needs of a patient, is of paramount importance. Pre-visualization of the available options can be achieved with the help of Digital Smile Design, in conjunction with the most effective esthetic parameters available: Bolton analysis, golden proportion, especially in cases of longer incisors, RED proportion, and also the formula used to simplify the tooth size proportions by using the lower anterior incisors as a reference. The predominant demographic of patients looking to correct their anomalous dentition is young people. Therefore, it is even more important to ensure we use as minimally invasive a treatment plan as possible in order to extend the longevity of the restoration, allowing the patient to benefit from the current treatment for a longer period before eventual re-treatment becomes necessary. It is important to remember that ideal orthodontic treatment options may be overestimated by clinicians when compared to laypeople’s smile perception. In cases of patients with both congenitally missing maxillary lateral incisors and a high smile line, that are undergoing orthodontic space closure, extrusion of the canine and intrusion of the first premolar is recommended for optimal gingival margin contouring. At the same time, the patient has shown themselves to be most concerned with the color and shape of the restoration, so always keeping in mind the primary concerns of the patient is advised.

## Figures and Tables

**Figure 1:**
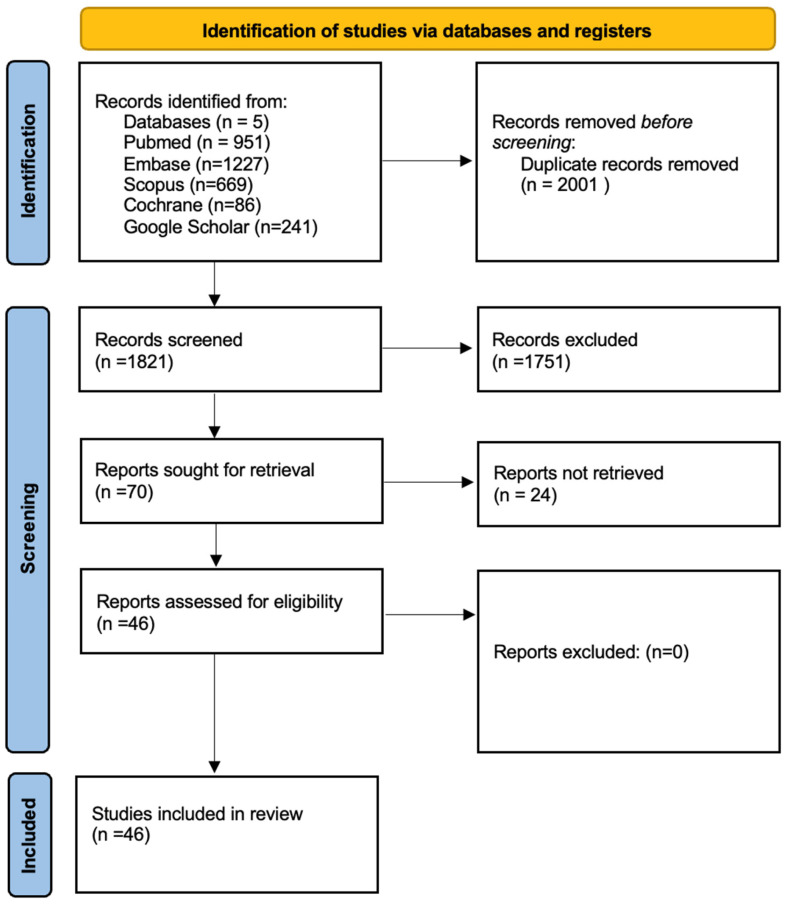
PRISMA flow diagram [19].

**Table 1:** Etiology, prevalence, and appearance of dental anomalies.

Anomaly Type	Name	Etiology	Prevalence	Appearance
**STRUCTURE**	Amelogenesis imperfecta	Inherited in an autosomal dominant manner and are caused by mutations in the FAM83H gene. Disturbance during apposition stage of dental development.	0.43%	-Hypoplastic—enamel may be thin or pitted [13] -Hypocalcified—enamel is dull, opaque white or brown-colored -Hypomaturation—mottled or frosty looking white opacities at incisal third of crown, “snow-capped teeth” appearance.
	Dentinogenesis imperfecta Type 2	Changes in the DSPP gene and is inherited autosomal-dominantly. Disturbance during apposition stage of dental development.	0.0125%	Opalescent brown or blue hue, bulbous crowns.
**SIZE**	Macrodontia	General enlargement of all teeth can be observed in certain endocrine conditions. It can be linked to a form of localized gigantism due to an excess of growth hormone. Disturbance during the bud stage of dental development.	0.03%	Anything above average size is considered macrodont -Central incisor 9 mm wide -Lateral incisor 7.5 mm wide
	Microdontia	Various environmental and genetic factors can cause the tooth to be underdeveloped. Disturbance during the bud stage of dental development.	1.51%	Teeth that are smaller than normal. It is distinguished by the reduction in the mesiodistal and cervico-incisal diameters (due to coronary alteration or level of the gingival margins) of the dental crown.
	Short roots	Various environmental and genetic factors can cause the root to be underdeveloped. Disturbance during bell stage of dental development.	1.3%	Seen on radiograph as significantly shorter than average.
**NUMBER**	Supernumerary-mesiodens	Disturbances during the initiation stages of dental development.	0.36%	Ref. [14] can be conically or tuberculate shaped, more usually seen as a single tooth, not uncommonly as a pair of extra teeth on the palatal side of crowns.
	Hypodontia-agenesis	The *PAX9* gene was frequently linked to a high risk of maxillary lateral incisor agenesis due to genetic familial inheritance. Disturbance during the initiation stage of dental development.	Europe (5.5%) Australia (6.3%) [15]	Missing tooth element.
**MORPHOLOGY**	Dens evaginatus (talon cusp)	DE arises during the bell stage of tooth development when some of the dental papilla’s inner enamel epithelium and adjacent ectomesenchymal cells proliferate and fold abnormally into the enamel orga’s stellate reticululm.	0.6% [16]	The morphology of an accessory cusp has been described as an abnormal tubercle or extrusion that protrudes above the neighboring tooth surface. Characterized by enamel covering a dentinal core that typically contains pulp tissue, occasionally with a thin pulp horn.
	Tooth fusion Double tooth/tooth gemination	The union of two normally separated tooth germs results in fusion (synodontia). One tooth bud attempting to divide into two crowns with a single root canal is known as gemination. During development, tooth buds may fuse or geminate. Disturbance is during the cap stage.	Gemination 0.07% Fusion 0.23%	In contrast to gemination, which produces teeth with a common pulp chamber, fusion produces teeth with distinct pulp chambers that unite at the dentin level. Radiological examination is the best way to differentiate between a fused or geminated tooth.
	Dens invaginatus	Arises from the dental papilla folding inward during tooth development [17]. Disturbance during the cap stage of development.	0.3%	The affected teeth exhibit a deep infolding that may extend deep into the root, beginning at the foramen coecum or the tip of the cusps.
	Peg-shaped laterals	Various environmental and genetic factors can cause the tooth to be underdeveloped [13]. Disturbance during the bud stage of dental development.	1.58%	Conical appearance
	Conoid teeth	Various environmental and genetic factors can cause the tooth to be underdeveloped. Disturbance during the bud stage of dental development.	0.6% [18]	Short clinical crowns often without contact points, known as tapered teeth.

**Table 2:** Inclusion and exclusion criteria.

	Inclusion Criteria	Exclusion Criteria
**Population/Patient/Problem**	Permanent dentition (apart from 3rd molars) Non-syndromic patients Dental anomalies in morphology, structure, size, and number **Structure** Amelogenesis imperfecta Dentinogenesis imperfecta (type 2) **Size** Macrodontia Microdontia Short roots **Number** Supernumerary/hyperdontia Agenesis Mesiodens (searched with supernumerary) **Morphology** Talon cusp—dens evaginatus in the anterior teeth Tooth fusion and gemination Dens invaginatus Peg-shaped laterals Conoid teeth Maxillary incisors—central and lateral incisor	Primary or mixed dentition patients Syndromic patients including, but not limited to: Ectodermal dysplasia Osteogenesis imperfecta Cleft lip and palate Gardner syndrome Turner’s hypoplasia Dentinogenesis imperfecta type 1 and 3 Type 1 is frequently associated with osteogenesis imperfecta Dental anomalies of position, such as impacted canines and ectopic teeth. MIH—pediatric cases Fluorosis and hypoplasia -Oligodontia—frequently associated with a syndromic disorder -Anodontia Trauma—dilaceration, subluxation, fracture Any tooth other than the maxillary incisors, except for maxillary canines, when used in canine mesialization as a conservative treatment for MLIA
**Intervention**	Restorative rehabilitation: Composite bonding Crowns Veneers Bridge (Maryland or cantilever) Space closure or expansion if agenesis Bleaching and microabrasion Crown lengthening procedure	Surgical rehabilitation (implants) Surgical intervention—autotransplantation, impaction, transmigration Endodontic treatment
**Comparison**	Conservative restorative techniques Anomalous vs. non anomalous dentition	Surgical techniques No treatment
**Outcome**	Primary outcome: Based on standardized esthetic indexes such as: Golden ratio Bolton analysis Smile Esthetic Index RED proportion Patients’ own satisfaction Secondary outcome: Quality of life Long term durability of the restorations	Non-quantifiable results
**Type of Study**	Randomized controlled trials Research studies Case reports Case–control studies Retrospective studies Literature reviews	Systematic reviews and meta-analyses Narrative reviews Letters to the editor In vitro studies

**Table 3:** ROBINS–I risk of bias assessment.

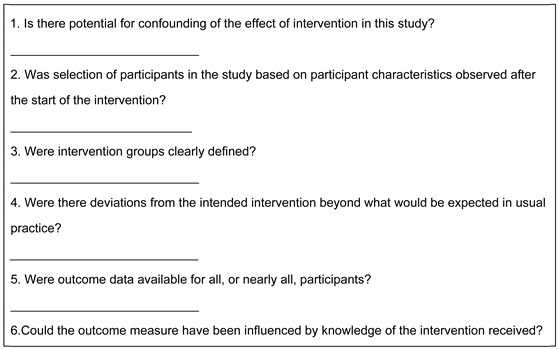								
+	low risk of bias							
-	high risk of bias							
x	unclear risk of bias							
**AMELOGENESIS IMPERFECTA + DENTINOGENESIS IMPERFECTA.**								
**Laverty, Dominic P [22]**	**2016**	**-**	**+**	**+**	**+**	**x**	**+**	**x**
Nathwani, N.S. and Kelleher, M. [23]	2010	-	**+**	**+**	**+**	**x**	**+**	**x**
Büchi, D. and Fehmer, V. [24]	2014	-	**+**	**+**	**+**	**x**	**+**	**x**
**CONOID TEETH.**								
**Pena, C.E. and Viotti. [25]**	**2009**	**-**	**+**	**+**	**+**	**x**	**+**	**x**
**HYPODONTIA.**								
**Ford, S., Ashley, M.P. [26]**	**2023**	**-**	**x**	**x**	**+**	**x**	**+**	**x**
**MICRODONTIA + MACRODONTIA.**								
**Waldman, A.B. [27]**	**2008**	**-**	**+**	**+**	**-**	**x**	**+**	**x**
German, D.S. and Chu, S.J. [6]	2016	**+**	**x**	**+**	**+**	**x**	**+**	**x**
Lopes-Rocha, L. and Garcez. [28]	2022	-	**+**	-	**+**	**+**	**+**	**x**
**MAXILLARY LATERAL INCISOR AGENESIS (MLIA).**								
**Brough E, et al. [29]**	**2010**	**+**	**+**	**x**	**-**	**+**	**x**	**x**
Thierens, L.A.M., Pet al. [30]	2017	**+**	-	**x**	**+**	-	**+**	**x**
Stylianou A, et al. [31]	2016	**+**	**+**	-	-	**+**	**+**	**x**
Qadri S, et al. [32]	2016	**+**	**+**	-	-	**+**	**+**	**x**
Mota, A. et al. [33]	2016	**+**	**+**	-	**+**	**+**	**+**	**x**
Pinho, T. et al. [34]	2015	**+**	**+**	-	-	**+**	**+**	**x**
Lombardo, L. et al. [35]	2014	**+**	**+**	-	-	**+**	**x**	**x**
Alkadhimi, A. et al. [36,37]	2022	**+**	**+**	-	-	**+**	**+**	**x**
Rayner, W.J. et al. [38]	2015	**+**	**+**	-	-	**+**	**+**	**x**
Araújo, E.A. et al. [39]	2006	**+**	**+**	-	**x**	**+**	**+**	**x**
Ulhaq, A. et al. [40]	2019	**+**	**+**	-	-	**+**	**+**	**x**
Priest, G. [41]	2019	**+**	**+**	-	-	**+**	**+**	**x**
De-Marchi, L.M. et al. [42]	2014	**+**	**+**	-	-	**+**	**+**	**x**
German, D.S. et al. [6]	2016	**+**	**+**	**+**	-	**+**	**+**	**x**
Lopes-Rocha, L. et al. [28]	2022	**+**	**+**	-	-	**+**	-	**x**
Pini, N.P. et al. [43]	2012	**+**	**+**	-	-	**+**	**+**	**x**
Benito, P.P. et al. [4]	2012	**+**	**+**	-	**x**	**+**	**+**	**x**
Kokich Jr., V.O. et al. [44]	2005	**+**	**+**	-	-	**+**	**+**	**x**
Kokich Jr et al. [5]	2005	**+**	**+**	-	-	**+**	**+**	**x**
**PEG-SHAPED LATERAL.**								
**Rathi S, et al. [7]**	**2022**	**+**	**-**	**-**	**+**	**-**	**x**	**x**
Dolan, S. et al. [45]	2023	**+**	**+**	-	-	**+**	**+**	**x**
Omeish, N. et al. Bosco, J. [46]	2022	**+**	-	**+**	**+**	-	**x**	**+**
Izgi, Ayna [47]	2005	**+**	**+**	-	-	**+**	**+**	**x**
Saatwika, et al. [48]	2020	**+**	**+**	-	-	**+**	**+**	**x**
da Cunha, L.F. et al. [49]	2018	**+**	**+**	-	-	**+**	**+**	**x**
Irmaleny, I. et al. [50]	2024	**+**	**+**	-	-	**+**	**+**	**x**
Zorba, Y.O. et al. [51]	2010	**+**	-	-	-	**+**	**+**	**x**
Scarpelli, A.C. et al. [52]	2008	**+**	**+**	-	**+**	-	**+**	**x**
Ittipuriphat, I. et al. [53]	2013	**+**	**+**	-	-	**+**	**+**	**x**
**PERCEPTION.**								
**Thomas M, et al. [54]**	**2011**	**+**	**+**	**-**	**-**	**+**	**+**	**x**
Agou SH, et al. [55]	2020	**+**	**+**	-	-	**+**	**+**	**x**
Cracel-Nogueira F, Pinho T. [56]	2013	**+**	**+**	-	-	**+**	**+**	-
Rosa, Olimpo. [57]	2013	**+**	**+**	**+**	-	-	**+**	**x**
Kokich, et al. [5]	2006	**+**	**+**	-	-	**+**	**+**	**+**
De-Marchi, L.M. et al. [42]	2014	**+**	**+**	-	-	-	**+**	**+**
Mota, Pinho [33]	2015	**+**	**+**	-	**+**	**+**	-	**x**
Florez, Rinchuse. [58]	2019	**+**	**+**	-	-	**+**	**+**	**x**
Parkin, Benson [32]	2016	**+**	**+**	-	-	**+**	**+**	**x**
Bukhary S.M., et al. [59]	2007	**+**	**+**	-	**+**	**+**	-	-
Souza RA, et al. [10]	2017	**+**	**+**	-	-	**+**	**+**	**x**

**Table 4:** Synthesis of results.

**Amelogenesis Imperfecta and Dentinogenesis Imperfecta.**							
**Title**	**Year**	**Authors**	**Study Design**	**Population**	**Intervention**	**Comparison**	**Outcome**
The prosthodontic pathway for patients with anomalies affecting tooth structure	2016	Laverty, Dominic P and Thomas, Matthew BM [22]	Clinical article	N/A	Patients with AI and DI frequently have veneers placed on their anterior teeth. They permit good esthetics whilst being relatively conservative of tooth tissue in comparison to crowns.	Various restorative options—resin bonding vs. veneers vs. crowns and onlays.	Although adhesion to teeth affected by AI and DI is less predictable, minimal preparation is necessary to preserve as much tooth tissue as possible.
Minimally destructive management of amelogenesis imperfecta and hypodontia with bleaching and bonding.	2010	Nathwani, N.S. and Kelleher, M. [23]	Case report	20-year-old female	Prosthodontics for MILA—cantilever on canines in this case, plus composite bonding to maintain spaces and stop mesial drifting.	Bleaching, gingivectomy, composite bonding and cantilever bridges on canines were used instead of the more invasive traditional veneer or bridge placement	Patient very pleased with final outcome
Minimally invasive rehabilitation of a patient with amelogenesis imperfecta.	2014	Büchi, D. and Fehmer, V. and Sailer, I. and Wolleb, K. and Jung, R. [24]	Case report	27-year-old female	Crown lengthening on to prep for veneers later. At home bleaching for. Period of 2 weeks followed up by microabrasion. Veneer placement to alter position and shape of teeth—silicon key as a guide for prep before fabrication.	Veneers to cover the extensive staining—bleaching and microabrasion improved the underlying tooth color, but bonding would not have been as effective in this case.	Patient was happy with the final treatment outcome. At a follow-up visit 18 months post-insertion, all the veneers looked well integrated without any discoloration of the margin, or chipping or fractures of the ceramic
**Conoid Teeth.**							
**Title**	**Year**	**Authors**	**Study Design**	**Population**	**Intervention**	**Comparison**	**Outcome**
Esthetic rehabilitation of anterior conoid teeth: comprehensive approach for improved and predictable results.	2009	Pena, C.E. and Viotti, R.G. and Dias, W.R. and Santucci, E. and Rodrigues, J.A. and Reis, A.F. [25]	Case report	22-year-old female	Gingivectomy of laterals, bleaching, silicone index and mockup. Luting, laminate veneers for laterals and composite bonding for central incisors.	Conservative veneer prep allows its translucency to render a natural appearance vs. Ultraconservative veneer prep preserves the available enamel for bonding, thus increasing the prognosis for long-term bonding success.	Patient was satisfied with outcome
**Hypodontia.**							
**Title**	**Year**	**Authors**	**Study Design**	**Population**	**Intervention**	**Comparison**	**Outcome**
The dental technician as a member of the hypodontia multidisciplinary team, with practical considerations for anterior restoration design	2023	Ford, S., Ashley, M.P. [26]	Discussion article	N/A—hypodontia patients	Design of a resin-bonded bridge with bridge wing design, taking into account the incisal edge and the occlusal surface and also the abutment of choice.	Optimal abutment tooth: orthodontically repositioned canines are prone to rotation during relapse. As a result, a cantilever lateral incisor bridge attached to a canine may relapse. An alternative is to replace both lateral incisor teeth with a single restoration using a bridge that is appropriately crafted from both maxillary central incisors.	Replacing the missing anterior requires clinicians and dental technicians to collaborate. Ideal tooth placement and the creation of space between natural teeth can be made possible by mutual understanding, allowing restorations to be used to produce acceptable functional and esthetic results.
**Microdontia and Macrodontia.**							
**Title**	**Year**	**Authors**	**Study Design**	**Population**	**Intervention**	**Comparison**	**Outcome**
Smile design for the adolescent patient--interdisciplinary management of anterior tooth size discrepancies	2008	Waldman, A.B. [27]	Discussion Article	Adolescents	Interdisciplinary diagnosis and treatment plan. Orthodontic space management. Vertical management of gingival margins 4) Restorative phase. 5) Finishing.	These clinical principles for treating adolescent patients with a significant TSD. Bolton analysis to determine tooth size discrepancies.	The most important factors in clinical planning: tooth size, tooth shape, tooth proportions, occlusion, incisal edge position, gingival margin position, restorative material, treatment timing and sequence.
Simplifying optimal tooth-size calculations and communications between practitioners	2016	German, D.S. and Chu, S.J. and Furlong, M.L. and Patel, A. [6]	Discussion Article	Orthodontic patient	Anomalous incisors can be restored early during orthodontic correction. At least 2 mm is created around the lateral incisors by the orthodontist. The brackets are taken out, the dentist resizes and shapes them to the patient’s specifications, the brackets are put back in, and the gaps are sealed.	New methods vs. Bolton Analysis—both have some discrepancies. In contrast to the normal anterior Bolton ratio of 77.2 percent, the tooth widths in this example yield a total tooth mass of 45 mm for the maxillary anterior teeth; 36 mm divided by 45 mm yields an anterior Bolton ratio of 80 percent.	Since the mandibular central incisor is the least variable of the 12 anterior teeth, its width can be used to determine the ideal sizes of the other teeth when several of them are abnormal, missing, or not of the proper size. As a result, the ideal maxillary incisor widths can be determined by measuring their width.
Maxillary lateral incisor agenesis and microdontia: Minimally invasive symmetric and asymmetric esthetic rehabilitation	2022	Lopes-Rocha, L. and Garcez, J. and Tiritan, M.E. and da Silva, L.F.M. and Pinho, T. [28]	Case Report	Adolescent orthodontic patients	Pretreatment planning using Bolton analysis and golden proportion. Bleaching, enameloplasty, and bonding with composite resin can enhance esthetics and functions following orthodontic space closure.	Following orthodontic treatment for the six anterior upper and lower teeth, an analysis of tooth movement using golden proportion and Bolton’s anterior was conducted prior to treatment planning.	An alternative method for figuring out how much room to make for an MLI is the Bolton analysis. The most efficient and esthetically pleasing results for patients are generally acknowledged to be obtained through a diagnostic wax-up and a smile simulation. The proportion in gold (i.e., E.) For figuring out the MLI width, a fixed ratio of 1.618:1 can serve as a framework.
**Peg Laterals.**							
**Title**	**Year**	**Authors**	**Study Design**	**Population**	**Intervention**	**Comparison**	**Outcome**
A Multidisciplinary Aesthetic Treatment Approach for Peg Lateral of the Maxillary Incisors	2022	Rathi S, Dhannawat P, Gilani R, Vishnani R. [7]	Case report	22 yr old female	Frenectomy and midline diiastema closure, space created for peg laterals’ build up in composite bonding.	Treatment options; canine mesialization and recontouring. Replacement fixed partial dentures (FPDs), restoration peg lateral incisors.	A multidisciplinary approach in treatment results in enhances a conservative yet very esthetic finished result.
Restorative dentistry clinical decision-making for hypodontia: peg and missing lateral incisor teeth	2023	Dolan, S. and Calvert, G. and Crane, L. and Savarrio, L. and P Ashley, M. [45]	Clinical article	N/A	Multidisciplinary; MLIA—space closure or opening. PEG—removal or retaining tooth with composite build up.	Space closure vs. opening in MLIA. Removal or retain peg lateral.	No mention
Esthetic and functional rehabilitation of peg-shaped maxillary lateral incisors: Practical recommendations	2022	Omeish, N. and Nassif, A. and Feghali, S. and Vi-Fane, B. and Bosco, J. [46]	Clinical case	N/A	Multidisciplinary: orthodontic movement and veneer on one peg lateral. Composite bonding on the other. Veneer on the left lateral due to its shape and the high interproximal space to fill. Direct composite on right lateral due to low interproximal space.	Veneer versus composite bonding. Digital Smile Design and mockup to help with orthodontic finishing and help with tooth preparation.	Digital Smile Design to determine the lateral incisor dimensions—during the orthodontic finishing phase. To facilitate the movement of the peg-shaped affected teeth, DSD can be transformed into a mock-up
Direct restorative treatment of peg-shaped maxillary lateral incisors with resin composite: A clinical report	2005	Ayca Deniz Izgi and Emrah Ayna [47]	Clinical report	4 adult patients	Direct resin composite laminate veneers	Resin composite bonding versus ceramic restorations.	Resin composite shown to be more conservative of the underlying dentition, less brittle as a material and less abrasive for the opposing dentition in comparison to its porcelain counterparts.
Esthetic correction of peg laterals—A case report	2020	Saatwika, L. and Anuradha, B. and Gold Pearlin Mary [48]	Case report	19-year-old female	Ortho treatment for midline diastema, correction of peg laterals using direct composites and putty technique	Advantages and disadvantages of composite bonding.	Good esthetics and good periodontal outcome.
Tooth movement with elastic separators before ceramic veneer treatment: Rearranging asymmetric diastemas by managing the horizontal distance	2018	da Cunha, L.F. and Gugelmin, B.P. and Gaião, U. and Gonzaga, C.C. and Correr, G.M. [49]	Case reports	18-year-old female	Space creation with interdental elastics orthodontic movement to increase the proximal spaces and redistribute the diastemas before closing with minimally invasive ceramic veneers.	Redistribution of spaces before veneer placement or not.	Revaluation of patients smile after 10 months after cementation—anterior tooth proportion, periodontal health and smile arc were all optimal.
Componeer as an aesthetic treatment option for anterior teeth: a case report	2024	Irmaleny, I. and Hidayat, O.T. and Handayani, R.A.P. [50]	Case report	32-year-old female	Use of componeer Digital Smile Design, wax-up and mockup used to determine ideal proportions.	Ceramic veneers versus composite	In order to attain a golden proportion, the final restoration results demonstrate an increase in each tooth’s height and width.
Direct laminate veneers with resin composites: two case reports with five-year follow-ups.	2010	Zorba, Y.O. and Bayindir, Y.Z. and Barutcugil, C. [51]	Case report	15-year-old female	Direct laminate resin-based composite veneers to improve esthetics.	Veneer vs. composite bonding. DSD and mockup help with orthodontic finishing and tooth preparation.	Very satisfied patient.
Seven-year follow-up of esthetic alternative for the restoration of peg-shaped incisors	2008	Scarpelli, A.C. and Reboucas, A.P.S. and Compart, T. [52]	Case report	14-year-old identical twins	Direct composite bonding of the right peg lateral in both twins.	The dimensions of the maxillary left lateral incisor served as reference.	Very satisfied patients.
Anterior space management: Interdisciplinary concepts	2013	Ittipuriphat, I. and Leevailoj, C. [53]	Discussion	N/A	CLP, minor tooth movement and bleaching prior to restoration. Composite bonding corrected anterior spacing. Final restoration of ceramic veneers on the lateral peg-shaped incisors.	Golden proportion vs. RED proportion.	RED proportion show need for gingival margin alteration and orthodontic movement. Tooth spacing analyzed based on the (RED) proportion.
**Perception.**							
**Title**	**Year**	**Authors**	**Study Design**	**Population**	**Intervention**	**Comparison**	**Outcome**
Perception differences of altered dental esthetics by dental professionals and laypersons	2011	Thomas M, Reddy R, Reddy BJ. [54]	Comparative study	Orthodontists vs. general dentists vs. laypersons	Three images of smiles were altered using a software-imaging program. The alterations involved the crown length, crown width, midline diastema, and the papillary height of the maxillary anterior teeth. These images were then rated.	All three groups could identify a unilateral crown width discrepancy of 2.0 mm. No group considered a small diastema in the midline to be unattractive. In general, people found it less appealing when papillary height was reduced.	When assessing asymmetric crown length discrepancies, orthodontists were more critical than general dentists and laypeople. Asymmetric alterations make teeth more unattractive not only to the dental professionals, but also to laypersons.
Dimensions of Maxillary Lateral Incisor on the Esthetic Perception of Smile: A Comparative Study of Dental Professionals and the General Population	2020	Agou SH, Basri AA, Mudhaffer SM, Altarazi AT, Elhussein MA, Imam AY. [55]	Comparative study	156 participants, 36 patients with hypodontia (HP), 54 non-hypodontia patients (NHP), and 30 dentists (D).	Using computer software, two sets of photos were produced with the maxillary incisor dimensions altered. The width of the maxillary lateral incisors was altered in the first set. Only the maxillary lateral incisor length was altered in the second set, while the gingival margins remained unchanged.	The smiles with a lateral incisor to central incisor width proportion of 77 percent, according to 25.0 percent (HP), 40.8 percent (D), and only 4.2 percent of (P), were the most attractive. The smiles with 52.0 percent (HP), 20.8 percent (P), and 49.0 percent (D) were the least popular.	Not all groups were thought to be the most attractive, including the golden proportion. Dentists may have different esthetic opinions than their patients. Not all maxillary lateral incisor width reductions were generally deemed acceptable.
Assessment of the perception of smile esthetics by laypersons, dental students and dental practitioners	2013	Cracel-Nogueira F, Pinho T. [56]	Comparative study	292 laypersons, 241 dental students and 101 practitioners	With Adobe Photoshop, a smile’s manipulated elements—the gingival exposure, gingival margin level, crown length, maxillary midline, and inter-incisor diastema—were changed. Visual analogue scale (VAS) responses were gathered and assigned a score between 1 and 10.	The medium smile was regarded as the most esthetic, and high smile line and diastemas the least. Importance of MCI in smiling shown via the preference for asymmetry of the gingival margin at the maxillary lateral incisors (MLI).	Professionals were more critical in their scoring, while laypeople, dental students, and dental professionals all had different opinions about how attractive various modified features were, with the exception of diastemas.
Smile attractiveness of patients treated for congenitally MLIA as rated by dentists, laypersons, and the patients themselves	2014	De-Marchi, L.M. and Pini, N.I. and Ramos, A.L. and Pascotto, R.C. [42]	Comparative study	60 patients with MLIA, 20 laypersons, and 20 dentists	Evaluation of smile attractiveness in patients treated with space closure or space opening.	Patients treated with space closure reported higher satisfaction with their smiles compared to those treated with space opening.	Laypersons rated smiles with space closure more attractive than space opening. Dentists had a higher rating for space opening.
Influence of maxillary lateral incisor width ratio on perception of smile esthetics among orthodontists and laypersons	2022	Daniela Martinez Florez [58]	Cross-sectional study	283 laypersons and 83 orthodontists	A smile showing the lips and gingival margins was selected. The smile was standardized for maxillary central incisor width proportions and ideally perceived smile esthetics. In symmetrical increments of the central incisor ratio, the maxillary lateral incisor width was adjusted from 4:10 to 8:10.	Evaluated the perception of smile esthetics with different width ratios of maxillary lateral incisors among laypeople and orthodontists.	For orthodontists, the most attractive width ratio was 5.7:10, conversely for was 8:10, although laypersons ranked all ratios very similarly. The width ratio of 4:10 was ranked lowest by both groups. When evaluating esthetics, orthodontists were more critical.
Space closing versus space opening for bilateral missing upper laterals—aesthetic judgments of laypeople: a web-based survey	2016	Parkin, Benson [32]	Web-based survey	959 participants including staff and students at the University of Sheffield	Evaluation of 10 images of space closing (OSC) versus prosthetic replacement (PR) in images of patients with missing upper lateral incisors. Using a 5-point Likert scale.	The mean rating for OSC images was 3 points 34 out of 5, which is higher than the mean rating for PR images, which is 3 points 14 out of 5. Three out of four paired images were preferred by both males and females as OSC images.	The general public found space closing more appealing than space opening. Higher ratings of attractiveness were typically given by female and staff judges.
The influence of varying maxillary lateral incisor dimensions on perceived smile aesthetics	2007	Bukhary SM, Gill DS, Tredwin CJ, Moles DR. [59]	Clinical study	41 hypodontia patients, 46 non-hypodontia ‘control’ patients, and 30 dentists.	Alteration of maxillary lateral incisor dimensions in images. Initially, the maxillary lateral incisors’ width in relation to the central incisor was changed. In a second group, the lateral incisor’s length was changed in increments of 0–5 mm.	Laypersons were more tolerant of variations in width ratios. All groups preferred the lateral to central width proportions of 67 percent and 72 percent, respectively. It was found that the most common maxillary lateral incisor length was 1–1.5 mm shorter than the central.	The golden proportion is a range rather than a single value. The 67% lateral-to-central width proportion followed by the 72% width proportion were most preferred. Very long + very short lateral incisors were thought to be the least attractive. Patients with hypodontia favored longer lateral incisors.
Esthetic perception of maxillary lateral incisor agenesis treatment by canine mesialization	2015	Antonino Mota, Teresa Pinho [33]	Observational study	654 participants including 303 laypersons, 215 general dentists, 55 prosthodontists, and 82 orthodontists.	Nine images were digitally modified from the same frontal intraoral photograph to show various treatment options for space closure in MLIA for comparison along with a questionnaire.	The MLIA restoration perceived to be most attractive showed unilateral dental and gingival reshaping. Dental and gingival reshaping was considered the most attractive whereas unmodified mesialization was considered the least attractive.	Regarding SC treatments, all groups regarded simple dental reshaping of the canine to be attractive, only the dental professionals considered gingival and crown reshaping to be the most esthetic.
Perceptions of dental professionals and laypeople to altered dental esthetics in cases with congenitally missing maxillary lateral incisors	2013	Marco Rosa, Alessia Olimpo, Rosamaria Fastuca and Alberto Caprioglio [57]	Observational study	160 participants including laypeople, adult orthodontic patients, general dentists, and orthodontists.	Alterations of dental esthetics in images (crown length, crown width, midline diastema, and papillary height) using software-imaging program. Professionals and laypeople were found to perceive smiles significantly differently.	Asymmetric alterations were deemed unattractive by both groups. Dental tipping and a noticeable diastema in the arch were discordant features that no one liked to see in a smile. Simulations associated with SC orthodontic treatment were ranked as the most attractive smile and significantly ranked higher by dental professionals than patients and laypeople.	Orthodontists were more critical of asymmetric crown length discrepancies. All groups identified a 2.0 mm crown-width discrepancy. Midline diastema was not rated unattractive. Papillary height reduction was generally rated less attractive. Treatment, absence of diastema, and symmetry were the most accepted characteristics by all.
Perceptions of dental professionals and laypersons to altered dental esthetics: Asymmetric and symmetric situations	2006	Vincent O. Kokich, Vincent G. Kokich, and H. Asuman Kiyak [63]	Observational study	General dentists, orthodontists, and laypersons.	A software imaging program was used to modify seven pictures of women’s smiles. The alterations involved crown length, crown width, midline diastema, papilla height, and gingiva-to-lip relationship of the maxillary anterior teeth. These altered images were rated using a visual analog scale.	Orthodontists were more critical than others when evaluating asymmetric crown length discrepancies. A unilateral crown width discrepancy of 2 points 0 mm was found by all groups. No group found a small midline diastema to be unattractive. Bilateral alteration was deemed more appealing than unilateral papillary height reduction.	In general, asymmetric alterations make teeth more unattractive to not only dental professionals but also the lay public. Symmetric alterations might appear unattractive to dental professionals, but the lay group often did not recognize some symmetric alterations.
Perception of attractiveness of missing maxillary lateral incisors replaced by canines	2018	Souza RA, Alves GN, Mattos JM, Coqueiro RDS, Pithon MM, Paiva JB. [10]	Observational study	150 laypersons and 100 dentists and dental students	A 20-year-old woman’s smiling front view extraoral photo was digitally modified to mimic agenesis and its treatment by shifting, bleaching, or reshaping the gingival and canine contours.	While dentists thought that MLIA with canine repositioning and reshaping was the least attractive, students and laypeople thought that MLIA with canine repositioning, gingival contour, bleaching, and reshaping was the worst.	Dentists and students were more likely to accept treatment approaches that involved altering the gingival contour than laypeople, who preferred approaches that merely involved reshaping.
**Maxillary Lateral Incisor Agenesis.**							
**Title**	**Year**	**Authors**	**Study Design**	**Population**	**Intervention**	**Comparison**	**Outcome**
Canine substitution for missing maxillary lateral incisors: the influence of canine morphology, size, and shade on perceptions of smile attractiveness	2010	Brough E, Donaldson AN, Naini FB. [29]	Observational study	120 participants (40 orthodontists, 40 dentists, and 40 laypeople)	A smiling photograph of a hypodontia patient who had OSC with maxillary canines replacing the lateral incisors. Image digitally altered the canine gingival height, crown tip height, canine width, and canine shade.	The most appealing canine gingival height was 0–5 mm below the maxillary central incisor’s gingival margin. It was thought that pointed canines, wider canines, and taller canine tips were unsightly.	Dark canines were ranked least attractive by all groups, Natural shades were preferred by dentists and brighter shades by orthodontists and laypeople. Narrow canine crowns frequently were ranked as most attractive.
An esthetic evaluation of unilateral canine substitution for a missing maxillary lateral incisor	2017	Thierens LAM, Verhoeven B, Temmerman L, De Pauw GAM. [30]	Observational study	174 examiners of orthodontists, periodontists, dentists, and laypeople	By modifying a photograph from the standard, the following parameters were investigated: (1) width, (2) color, (3) gingival margin height, (4) crown tip morphology of the substituted canine, and (5) gingival margin height of the neighboring first premolar.	Overall, a darker canine color and a more pronounced canine tip morphology were significantly ranked as most unattractive (*p* < 0.05). The gingival height of the neighboring premolar was ranked as least unattractive by all groups of examiners.	Darker canine color and a pronounced tip morphology of a substituted canine are rated as the most unattractive by dental professionals and laypeople.
Restoring Congenitally Missing Maxillary Lateral Incisors Using Zirconia-Based Resin Bonded Prostheses	2016	Stylianou A, Liu PR, O’Neal SJ, Essig ME. [31]	Clinical report	17-year-old patient	For a young patient with very high esthetic standards, zirconia-based resin-bonded fixed partial dentures (RBFPDs) were chosen as a practical and conservative treatment option. After being digitally designed, the bilateral-winged zirconia frameworks were milled with (CAD/CAM)-controlled milling machine.	Tooth preparation design and subsequent RBFDP framework design are fundamental for the mechanical retention and strength of the prosthesis. An increased frequency of adhesive debonding has been recorded for non-retentive prepared RBFDP retainers.	Completion of the treatment resulted in a functional and esthetic successful outcome and a 17-month follow-up presented uneventful. Esthetic expectations and potential ongoing growth of the patient must be thoroughly considered.
Esthetic Assessment of the Effect of Gingival Exposure in the Smile of Patients with Unilateral and Bilateral Maxillary Incisor Agenesis	2015	Pinho, T. and Bellot-Arcís, C. and Montiel-Company, J.M. and Neves, M. [34]	Observational study	381 people (80 orthodontists, 181 general dentists, 120 laypersons)	In four cases, patients were asked to score the smiles’ attractiveness both before and after treatment, including two cases with bilateral MLIA and two cases with unilateral MLIA and contralateral microdontia. A computer was used to modify the buccal photo in each instance, applying standard lips to produce high, medium, and low smiles.	Photos of people with medium smiles scored higher than those with high or low smiles in both the pre- and post-treatment cases, and the differences were substantial. In all cases, orthodontists were the least tolerant evaluation group (assigning lowest scores), followed by general dentists.	The medium-height smile was considered to be more Symmetrical treatments scored higher than asymmetrical treatments. Gingival exposure had a significant influence on the esthetic perception of smiles in post-treatment cases.
Optimal parameters for final position of teeth in space closure in case of a missing upper lateral incisor	2014	Lombardo, L. and D′Ercole, A. and Latini, M.C. and Siciliani, G. [35]	Case–control	30 patients with MLIA	Space closure setup in the upper lateral incisor region was carried out in 30 individuals with agenesis in this specific tooth. The tip, torque, and in–out measurements were taken and contrasted with the data from earlier researchers.	With the exception of the first premolars, which need a larger tip, and the first molars, which need a smaller tip, the tip values in the upper dentition were similar to those reported by Andrews (Am J Orthod 62 (3):296–309, 1972).	An important suggestion, seen in other studies, is extruding the canine and intruding the first premolar in order to obtain ideal gingival architecture.
Camouflaging the permanent maxillary canine to substitute an absent lateral incisor—part 2: challenges and solutions	2022	Alkadhimi, A. and Razaghi, N. and Elder, A. and DiBiase, A.T. [37]	Clinical article	N/A	Some of the common problems in canine camouflage cases are discussed, along with potential fixes.	Bolton’s analysis is used to identify any disparity in tooth size. Gingival discrepancy: Intrusion and extrusion of the involved teeth, as well as a higher gingival margin, can change the gingival contour.	In general, the golden proportion concept of esthetics can serve as a helpful guide to attain ideal dimensions and symmetry. However, it is important to consider the patient’s perspective and acknowledge that there may be genetic, ethnic, and cultural differences in determining what is esthetically pleasing.
The effect of canine characteristics and symmetry on perceived smile attractiveness when canine teeth are substituted for lateral incisors	2015	Rayner, W.J. and Barber, S.K. and Spencer, R.J. [38]	Prospective cross-sectional study	90 participants (30 orthodontists, 30 dentists and 30 laypeople)	Different dentitions were displayed using a picture of a smiling woman. There was a control picture with the “ideal” smile, and six more pictures that replaced the maxillary lateral incisors with canine teeth of different sizes, either unilaterally or bilaterally.	Orthodontists and GDP rated smiles with canine substitution for lateral incisor agenesis to be significantly less attractive than an ideal smile unless the substituted canine teeth approximated the lateral incisor in terms of size, shape, color, and gingival margin. Regardless of the canine tooth characteristics, laypeople did not find the same smiles to be noticeably more or less attractive than an ideal smile. All groups did not find smiles with unilateral canine substitution to be noticeably less attractive than those with bilateral canine substitution.	Dental professionals were significantly more perceptive than lay people to the deviation from ideal smile esthetics when canine teeth were substituted for lateral incisors. Orthodontists, general practitioners, and laypeople did not find smiles with a unilaterally replaced canine tooth inherently less attractive than those with a bilaterally substituted canine tooth.
Diagnostic protocol in cases of congenitally missing maxillary lateral incisors.	2006	Araújo, E.A. and Oliveira, D.D. and Araújo, M.T. [38]	Literature Review	N/A	Discussion of clinical variables to be considered -canine position, color, functional occlusion and gingival height.	Canine color—the presence of color difference has a big impact on perception. Functional occlusion—can be obtained with lateral group function after space closure, as opposed to canine. Gingival height—Ideal anterior gingival architecture has the central incisor and canine margins at the same level, while the lateral incisor gingival contour is approximately 1 mm more incisal.	Canine position and its root angulation may be a complicating factor when the clinician decides to open space for a prosthesis. In patients with congenital MLIA the canines frequently show a mesial pattern of eruption. Such a condition favors using the canine as a substitute for the lateral incisor.
Dental Factors Influencing Treatment Choice For Maxillary Lateral Incisor Agenesis: A Retrospective Study	2019	Ulhaq, A. and Fee, P. and Cresta, M. and Turner, S. and Dutta, A. [40]	Retrospective study	44 MLIA patients previously treated	Baseline data consisted of patient records, pre-treatment orthodontic study casts and clinical photographs	Space closing versus space opening. Space opening importantly, the amount of bone that is available at the lateral incisor site—often found to be deficient. Space closing—surveys have shown that patient who have space closure and canine recontouring are perceived as having the best esthetic results.	Orthodontic space opening for MLIA is linked to the maxillary arch’s sufficient space.
The treatment dilemma of missing maxillary lateral incisors-Part I: Canine substitution and resin-bonded fixed dental prostheses	2019	Priest, G. [41]	Review article	N/A	Discussion of treatment options for MLIA Canine substitution and resin-bonded fixed dental prosthesis.	Canine substitution versus resin-bonded bridge. Multiple studies have shown canine substitution to be preferable esthetically compared to prosthodotic interventions, as well as preferable outcomes in terms of periodontal health. A RBFDP can be removed whenever it is no longer required with little to no modification of the adjacent teeth, and can be rebonded or remade again with minimal adverse effects on the supporting teeth.	Available data indicates that canine substitution and RBFDPs have demonstrated successful results and patient satisfaction. Dental teams should inform patients about the alternatives and mutually agree on the treatment solution tailored for each patient that provides the best potential for long-term esthetics and function.
Simplifying optimal tooth-size calculations and communications between practitioners	2016	German, D.S. and Chu, S.J. and Furlong, M.L. and Patel, A. [6]	Clinical article	N/A	A simple formula to determine optimal tooth sizes, along with an esthetic guide worksheet to use with collaborating dentists.	This method uses the mandibular incisors as a reference for size, especially in the anomalous dentition. The width of the mandibular central incisor can be used to calculate the ideal sizes of the other, anomalous dentition as it is the least variable tooth among the 12 anterior teeth.	Due to maxillary and mandibular teeth being smaller than normal in patients missing one or both maxillary lateral incisors space created or remaining for the final restorations may be smaller than ideal. Thus, clinicians should plan accordingly.
Maxillary lateral incisor agenesis and microdontia: Minimally invasive symmetric and asymmetric esthetic rehabilitation	2022	Lopes-Rocha, L. and Garcez, J. and Tiritan, M.E. and da Silva, L.F.M. and Pinho, T. [28]	Clinical study	40 orthodontic patients treated for MLIA	Casts were made and teeth were measured with a digital caliper at their greatest mesiodistal width and then compared with those of a control group matched for ethnicity, age, and sex.	The maxillary arch had a larger tooth size difference between the control and test groups than the mandibular arch maxillary and mandibular teeth are smaller than normal in patients missing one or both maxillary lateral incisors.	Because maxillary and mandibular teeth are smaller than normal in patients missing one or both maxillary lateral incisors space created or remaining for the final restorations may be smaller than ideal.
Fiber-reinforced framework in conjunction with porcelain veneers for the esthetic replacement of a congenital MILA	2012	Benito, P.P. and Trushkowsky, R.D. and Magid, K.S. and David, S.B. [4]	Case study	28-year-old female	For teeth 5–12, porcelain veneers and the congenitally missing upper right lateral incisor were replaced with a metal-free, two-component, resin-bonded Encore bridge.	Comparing the Encore bridge’s less invasive preparation to that of conventional metal ceramic full-coverage fixed partial dentures.	The metal-free RBFPD has proven to be a good substitute for single-tooth replacement in the anterior esthetic zone.
Managing congenitally missing lateral incisors. Part I: Canine substitution	2005	Kokich Jr., V.O. and Kinzer, G.A. [5]	Review article	N/A	Discussion of the most important parameters to consider for canine substitution, including malocclusion, profile, color, and shape of the canines and lip level. Malocclusion: most suitable for canine substitution—Angle Class II malocclusion with no crowding in the mandibular arch OR Angle Class I malocclusion with sufficient crowding to necessitate mandibular extractions.	Profile: generally, a balanced, relatively straight profile is ideal. Shape and color of canine; once too much does not have to be removed the shape can be modified, the color can be adjusted by bleaching and if not adequate, veneers. Smiling lip level; Positioning the natural canine’s gingival margin slightly incisal to the central incisor gingival margin is recommended.	Patient selection depends on the type of malocclusion, profile, the shape and color of the canines, and smiling level of the lips. These selection criteria must be evaluated prior to treatment in order to guarantee both treatment success and consistent esthetics.
Managing Congenitally Missing Lateral Incisors Part 2: Tooth-Supported Restorations	2005	Kokich Jr and Kinzer [44]	Review article	N/A	Discussion of the most important parameters to consider for Fixed Partial Denture and a comparison between Resin Bonded, Full Coverage or Cantilevered fixed partial denture	The most conservative tooth-supported restoration is the resin-bonded fixed partial denture, followed by the Cantilever fixed partial denture. The least conservative of all is a conventional full-coverage fixed partial denture.	Many restorative options exist. Depending on the type of final restoration that is chosen, interdisciplinary management of patients with congenitally missing lateral incisors often plays a vital role in the facilitation of treatment.
**TALON CUSP AND GEMINATED TEETH**							
**Title**	**Year**	**Authors**	**Study Design**	**Population**	**Intervention**	**Comparison**	**Outcome**
Bilateral geminated teeth with talon cusps: A case report	2012	Sener, S. and Unlu, N. and Basciftci, F.A. and Bozdag, G. [65]	Case report	Adolescent 17 yr boy	Talon cusps in both incisors were gradually reduced on two consecutive sittings held 6–8 weeks apart. The distinct enamel grooves running buccolingually on both central incisors were restored with a composite resin, and the esthetic appearance of anterior teeth was improved.	The purpose of the waiting period for cuspal reduction is to allow for the deposition of reparative dentin for pulpal protection and to avoid pulpal exposure. After both grinding procedures, the exposed surface was treated with fluoride gel as a desensitizing agent.	Patient was satisfied with the outcome.
Management of a facial talon cusp on a maxillary permanent central incisor: a case report and review of the literature	2014	Yazıcıoğlu, O. and Ulukapı, H. [62]	Case Report and Review of Literature	21 yr old Female	Minimal restorative treatment. The talon cusp was gradually reduced with a high-speed hand piece. The cusp was nearly completely eliminated without causing pulpal exposure. Direct resin-based composite was used.	Generally, the talon cusp area is grinded until reaching healthy tooth tissue. For this purpose, the labial surface of the tooth is painted with articulating paper. The purpose of this process is to ensure careful grinding.	Satisfied patient.

## Supplementary Materials

The following are available online at https://www.mdpi.com/article/10.3390/bioengineering12030262/s1, Table S1: PRISMA CHECKLIST.
